# Adaptation of the gut pathobiont *Enterococcus faecalis* to deoxycholate and taurocholate bile acids

**DOI:** 10.1038/s41598-022-12552-3

**Published:** 2022-05-19

**Authors:** F. Repoila, F. Le Bohec, C. Guérin, C. Lacoux, S. Tiwari, A. K. Jaiswal, M. Passos Santana, S. P. Kennedy, B. Quinquis, D. Rainteau, V. Juillard, S. Furlan, P. Bouloc, P. Nicolas, A. Miyoshi, V. Azevedo, P. Serror

**Affiliations:** 1 Université Paris-Saclay, INRAE, AgroParisTech, Micalis Institute, 78350 Jouy-en-Josas, France; 2 Université Paris-Saclay, INRAE, MaIAGE, 78350 Jouy-en-Josas, France; 3 Universidade Federal de Minas Gerais, ICB/UFMG, Minas Gerais, Belo Horizonte, 31270-901 Brazil; 4 Department of Computational Biology, USR3756 CNRS, Institut Pasteur, Paris, France; 5 Université Paris-Saclay, INRAE, MGP, 78350 Jouy-en-Josas, France; 6 Sorbonne Université, Inserm, Centre de Recherche Saint-Antoine, CRSA, AP-HP, Hôpital Saint Antoine, 75012 Paris, France; 7 Université Paris-Saclay, CEA, CNRS, Institute for Integrative Biology of the Cell (I2BC), 91198 Gif-sur-Yvette, France; 8 Present Address: Université de Strasbourg, INRAE, SVQV UMR-A 1131, 68000 Colmar, France; 9 Present Address: ImmunoSearch, Les Cyclades, Chemin de Camperousse, 06130 Grasse, France

**Keywords:** Microbiology, Molecular biology

## Abstract

*Enterococcus faecalis* is a natural inhabitant of the human gastrointestinal tract. This bacterial species is subdominant in a healthy physiological state of the gut microbiota (eubiosis) in adults, but can become dominant and cause infections when the intestinal homeostasis is disrupted (dysbiosis). The relatively high concentrations of bile acids deoxycholate (DCA) and taurocholate (TCA) hallmark eubiosis and dysbiosis, respectively. This study aimed to better understand how *E. faecalis* adapts to DCA and TCA. We showed that DCA impairs *E. faecalis* growth and possibly imposes a continuous adjustment in the expression of many essential genes, including a majority of ribosomal proteins. This may account for slow growth and low levels of *E. faecalis* in the gut. In contrast, TCA had no detectable growth effect. The evolving transcriptome upon TCA adaptation showed the early activation of an oligopeptide permease system (*opp2*) followed by the adjustment of amino acid and nucleotide metabolisms. We provide evidence that TCA favors the exploitation of oligopeptide resources to fuel amino acid needs in limiting oligopeptide conditions. Altogether, our data suggest that the combined effects of decreased DCA and increased TCA concentrations can contribute to the rise of *E. faecalis* population during dysbiosis.

## Introduction

The gastrointestinal (GI) tract shelters a complex community of hundreds of bacterial species, the GI-microbiota, that interacts intimately with the host. In balanced conditions, the physiological status of this ecosystem is termed ‘eubiosis’. The GI-microbiota plays a crucial role in maintaining eubiosis and preserving host health. Reciprocally, the lifestyle of the host modulates the composition of the gut microbiota and can disrupt the intestinal homeostasis, a physiological state called "dysbiosis"^1–5^. Antibiotics are notable compounds able to induce dysbiosis: They affect growth and viability of diverse species in the microbiota, vacating niches that may then be subject to overgrowth conducive to the development of infections by species so-called pathobionts^6,7^. Pathobionts are commensals with pathogenic potential, capable of adapting to dysbiosis and overgrowing, which can lead to opportunistic infections^1,7,8^.

Dysbiosis is characterized by changes in the nature and the concentration of many molecules in the GI environment. Among those, the bile acid composition of the GI-circulating bile is strongly affected. Bile acids (BAs) are amphipathic molecules ensuring crucial digestive functions and prominent antimicrobial barriers that enteric bacteria have to face. The nature and the concentration of BAs result from the interplay between the host and the GI-microbiota^9–13^. BAs are cholesterol-derived molecules synthesized in the liver through sequential steps resulting in two prominent primary BAs in humans, cholate (CA) and chenodeoxycholate (CDCA). CA and CDCA are then conjugated to amino-acids taurine or glycine, and the mixture is collected in the gallbladder before reaching the small intestine. One prominent primary-conjugated BA is taurocholate (TCA), synthesized from CA and taurine^14,15^. Before bile reaches the colon, ~ 95% of BAs is reabsorbed and returns to the liver. The remaining primary- and conjugated BAs in the gut are substrates for the GI-microbiota that performs de-conjugation (via bacterial bile salt hydrolases) and dehydroxylation (via bacterial 7, α-dehydroxylases) reactions generating secondary BAs, among which lithocholate (LCA) from CDCA, and predominantly, deoxycholate (DCA) from CA^9,16–19^. As a result of the GI-microbiota reshaping, dysbiosis hinders the ‘colonic-step’, i.e., de-conjugation and dehydroxylation reactions, and causes the increase in primary-conjugated BAs. Thus, the secondary BA, DCA, is a marker of eubiosis, whereas the primary-conjugated BA, TCA, is a marker of dysbiosis^9,14,15,20–25^.

*Enterococcus faecalis* is a bacterial species naturally present in the GI-microbiota in humans and vertebrates. In humans, under intestinal eubiosis conditions, *E. faecalis* is a subdominant species. Upon intestinal dysbiosis, *E. faecalis* is a pathobiont: it overgrows and can cross the intestinal barrier^26–28^, provoking infections mainly in elderly or immunocompromised patients^29–32^. How *E. faecalis* adapts to changes in BA composition during dysbiosis is a relevant question for the understanding of the intestinal overgrowth and pathogenesis. Analyses of gene expression in response to bile or BA mixtures, in *E. faecalis*^33–35^, and in other species^10,17,36,37^, reveal that bacteria undergo both a toxic effect and a cell-signaling effect. This is evinced by changes in the expression of genes involved in oxidative stress, detoxification and protein quality control, and central metabolic pathways related to fatty acid and phospholipid biosynthesis. While these single-timepoint snapshots are informative, the dynamics and complex mixture of bile, and the fact that specific bile-components can modulate subsequent adaptations of bacterial physiology, call for targeted examinations of bile compounds^10,37^. For instance, the natural incorporation of exogenous fatty acids present in bile into the cytoplasmic membrane of *E. faecalis* protects the bacterium against the membrane homeostasis-disrupting antibiotic daptomycin^38^. Secondary BAs, DCA and LCA impair separation of multiplying *E. faecalis* and *Enterococcus faecium* and disadvantage gut colonization by spontaneous DCA- or LCA-resistant mutants^39^. Also, changes in bile composition can modulate gut colonization by the bacteria; the toxic properties of CA and DCA are attenuated by the teichoic acid decorations of *E. faecalis* cell wall polysaccharides^40–43^.

The aim of this study was to better understand how *E. faecalis* adapts to changes in BA composition occurring in the gut, by analyzing its response to DCA, prominent during eubiosis and TCA, prominent during dysbiosis. We examined the growth pattern of *E. faecalis* in the presence of each BA and recorded a dynamic view of gene expression reprogramming that occurs within the first bacterial generation responding to DCA and TCA. Transcriptomic and physiological data showed that DCA impairs *E. faecalis* growth by altering the coordinated expression of essential genes. TCA has been shown to activate an oligopeptide importer, which promotes the growth of *E. faecalis* in amino-acid-limited conditions.

## Results and discussion

### *E. faecalis* is sensitive to DCA and resistant to TCA

**Figure 1:**
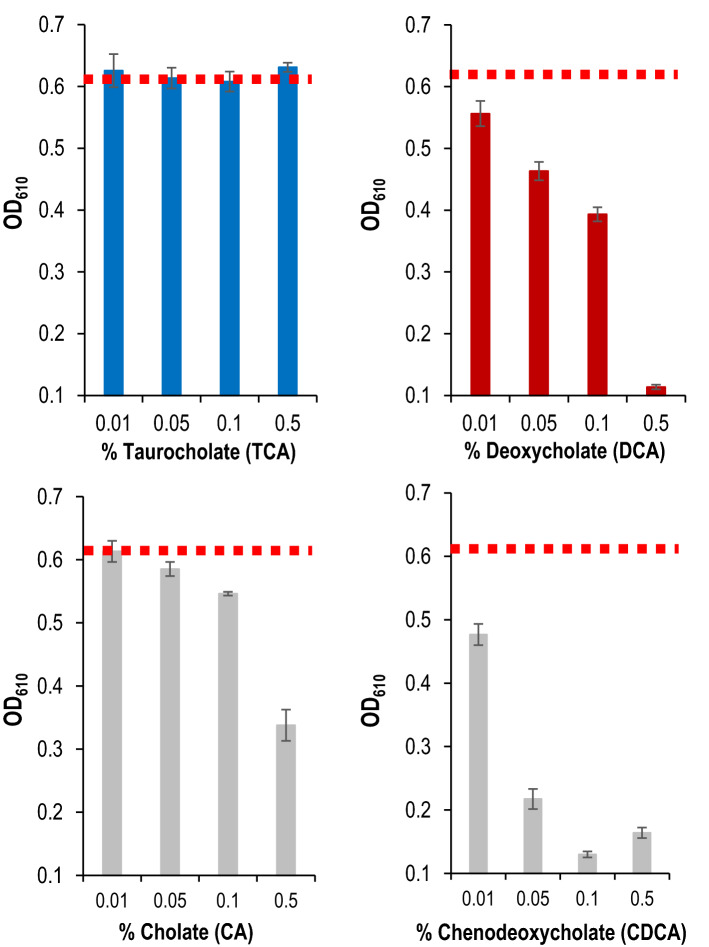
BA effects on *E. faecalis* growth. Mean values of the OD_610 nm_ measured 4 h after the addition of the BA to an exponentially growing culture. The growth control corresponded to the OD_610_ measured when BAs were replaced with water (marked by the horizontal dashed red line). The BA utilized for each set of experiments and its final concentration are indicated at the bottom of histograms. Mean values of the OD_610_ measured over 8 h growth after the addition of BAs are provided in Fig. S1. Error bars represent the standard deviation of the mean value calculated from at least three independent biological replicates performed in technical duplicates.

As amounts and the nature of each BA cannot be controlled experimentally in vivo, effects of major BAs (CA, CDCA, TCA and DCA) on the growth of *E. faecalis* have been evaluated in vitro and in BHI medium, at concentrations comparable to those found in the gut (from 0 to 0.5%)^44,45^. As for other bacterial species, specific growth effects were observed (Fig. 1, S1)^39,44,46,47^. The most severe growth impairments were observed with the most hydrophobic BAs^12^: CDCA and DCA at concentration as low as 0.01% (~ 240 µM). At 0.5%, these BAs totally inhibited growth. In comparison, 0.5% CA, a primary BA, had a less pronounced growth inhibition. In this range of concentration, TCA did not show any growth effect, a result in line with a previous report^47^.

The resistance of *E. faecalis* to TCA is remarkable, as TCA was shown to have toxic effects on other bacterial species at similar concentration ranges, *e.g., Escherichia coli*^47^, *Staphylococcus aureus*^46^, *Lactobacillus salivarius*^48^, or *Ruminococcus bromii*^44^. These species harbour genes encoding bile salt hydrolases (BSHs), which hydrolyse taurine- and/or glycine-conjugated BAs into their primary BAs (CA and CDCA). BSHs act with varying specificity and the sensitivity of bacterial species to TCA may result from the toxic effect of TCA-to-CA transformation. This hypothesis is further supported by data on BSH effects in *Lactobacillus acidophilus* and *Lactobacillus gasseri*^49^. The *E. faecalis* strain used in this study, a V583-derivative, has two BSH encoding genes, *ef0521* and *ef3005*^50^. In the growth conditions used, both genes are highly expressed^51^. Since no growth defects were observed, this suggests that neither of the *E. faecalis* BSHs hydrolyse TCA into CA (Fig. S1); an observation consistent with previous data showing that Ef0521 hydrolyses only glycine-conjugated BAs^52^.

Given that the intestinal environment can harbour a complex mix of all BAs, coupled with the observation that TCA does not affect growth of *E. faecalis*, we tested whether the deleterious effects of DCA could be mitigated by the presence of TCA. The growth impairment observed with DCA alone was not affected by the presence of 0.1% TCA, a physiological concentration, suggesting that the actions of these BAs do not interfere (Fig. S2).

### DCA affects vital functions in *E. faecalis*

To gain insight on how *E. faecalis* adjusts to the prominent BAs of the intestinal physiology, DCA and TCA, gene expression responses were analysed measuring evolving transcriptomes over short, sub-generation time, periods, in order to capture the chronology of gene expression remodelling in bacteria that sense and respond to the signal^53^. Bacteria were exposed to a low concentration of DCA (0.01%) to preserve growth as much as possible (Fig. S1). During the 15 minutes (min) of DCA treatment, the expression of ~ 900 CDSs were significantly changed, i.e., almost one third of CDSs in the chromosome (Tables S1, S2). We refer to any transcript as ‘CDS’, for a coding DNA sequence, i.e., portions of transcribed DNA that code for proteins or non-protein encoding RNAs (sRNAs, tRNAs, rRNAs). We analyzed changes in RNA levels and the chronology of functions involved in early [from zero to six minutes (t_6_0_)] and late responses [from six to fifteen min (t_15_6_)] (see methods). The transcriptome showed striking aspects: i) At all experimental time points, DCA affected the expression of CDSs known to be essential or important for growth, suggesting that *E. faecalis* was unable to establish an efficient steady state for the expression of its vital functions; ii) DCA disrupted the expression of many components of the translation machinery. Most likely these transcriptomic features are the most relevant in explaining the growth defect caused by DCA.

**Figure 2:**
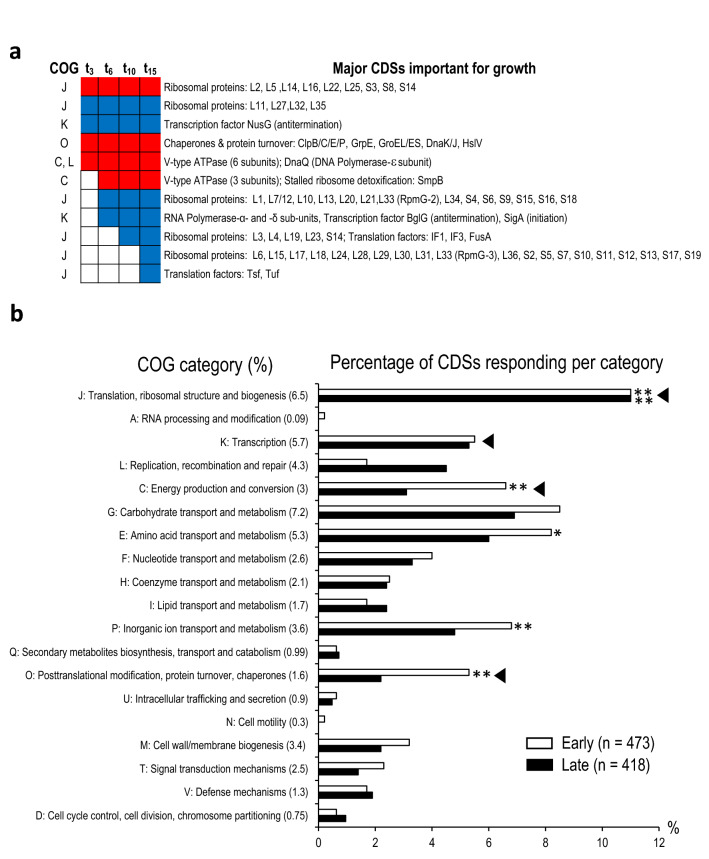
Examples of essential CDSs and COG functional categories reprogrammed during the adaptative response of *E. faecalis* to DCA. (**a**) Major CDSs encoding proteins essential or important for growth, responding to 0.01% DCA. Functions encoded by CDSs and experimental time points are listed on the right and at the top of the panel, respectively. COG assigned categories are noted on the left side of the figure by the letter code. The color code used for changes in RNA levels are: ‘red’, increase, ‘blue’, decrease; ‘white’, no change measured compared to the steady state level measured at t_0_. (**b**) COG functional categories reprogrammed in early (t_6_0_: White bars) and late (t_15_6_: Black bars) phase responses. COG categories are indicated on the left side of the histogram. For each category, the letter code is provided and the number in brackets indicates the percentage of CDSs within the entire chromosome assigned to the corresponding category, out of 3345 total CDSs (Table S3). For simplicity, categories ‘poorly characterized’ (i.e., COG categories ‘R’, ‘S’, and ‘-’, in Table S3) are not presented. Arrow heads indicate categories where changes in RNA levels for essential or important genes for growth have been measured in both early and late time intervals. For each time interval, ‘n’ corresponds to the number of CDSs that showed significant differential RNA levels. Significant COG enrichments are marked by * and ** corresponding to adjusted p-values ≤ 0.05 and ≤ 0.005, respectively.

DCA affected the RNA levels of 52 out of 61 ribosomal proteins, 26 out 68 tRNAs, translation initiation and elongation factors (IF1, IF3, FusA, Tsf, Tuf) (Fig. 2a, and Table S2). SmpB, an essential factor involved in the detoxification of stalled ribosomes was also significantly altered, further highlighting the effect on cellular translational machinery. Indeed, the COG category ‘J’ genes (‘translation, ribosomal structure and biogenesis’), 6.5% of CDSs in the genome, accounted for 11% of genes modified in both early and late phase response (Fig. 2b, and Table S3). Within the first three minutes, DCA provoked both increases and decreases in RNA levels encoding more than ~ 20% of ribosomal proteins (Fig. 2a), likely resulting in the discoordination of ribosomal component balance that is crucial to ensure ribosome assembly and translation efficiency^54,55^. Once this discoordination occurred, RNA levels dropped for near all the remaining ribosomal proteins (Fig. 2a and Table S2). This reprogramming occurred within 15 min in bacteria that sensed and responded to DCA. The tight coordination between functional ribosomes and growth rate^56–58^ strongly suggests that early discoordination and subsequent decreased of many ribosomal proteins substantially hinders the growth of subsequent generations of *E. faecalis* exposed to DCA.

Additional vital functions for growth were also affected by DCA: DNA replication, transcription machinery, folding and protein quality control (proteases and chaperones), pH homeostasis and membrane-potential (via the nine subunits of the essential type-V ATPase), (Fig. 2a)^57,59^. These observations are in line with reports indicating that a majority of these CDSs are also affected by bile or a mixture of BAs, in conditions that impair *E. faecalis* growth^33–35^. DCA also affects various environmental stress response functions, such as oxidative stress resistance (*sodA, ahpFC, npr*, *ohr*)^32^, chlorhexidine (*ef0576/79, ef2698*) and daptomycine responses (*liaXYZ*)^60,61^ (Table S2).

Many CDSs responding to DCA encode proteins known to be unfolded and aggregated by DCA-oxydation of their disulfide bonds: ribosomal proteins, chaperones and proteases^62^. Changes in RNA levels may reflect cellular attempts to preserve vital functions damaged by DCA. This process is expected to act while DCA is present and would imply dynamic adjustment to compensate for ongoing protein denaturation. Thus, the response to DCA has many characteristics that would explain growth impairment in addition to the large-scale dysregulation of cellular translational machinery (Fig. 1, 2).

Finally, DCA increased the expression of four loci known to be involved in virulence, biofilm development, adherence to epithelial cells and/or translocation^31^: In the early response, the *bopABCD* operon and *ebpC,* from the *ebpABC* operon; in the late phase, operons *fsrABDC-gelE-sprE* and *gls24/B* (Table S2). It is unclear why harsh growth conditions (i.e., eubiosis) stimulate the expression of factors involved in virulence. These genes may participate in stress adaptation or defence processes, such as *gls24/B*, known to contribute to bile salt resistance^63^, or *gelE* and *sprE*, which encode secreted proteases that inactivate antimicrobial peptides from the host or other microbiota species^64,65^.

### TCA signals a specific response for amino-acids and nucleotides metabolisms

**Figure 3:**
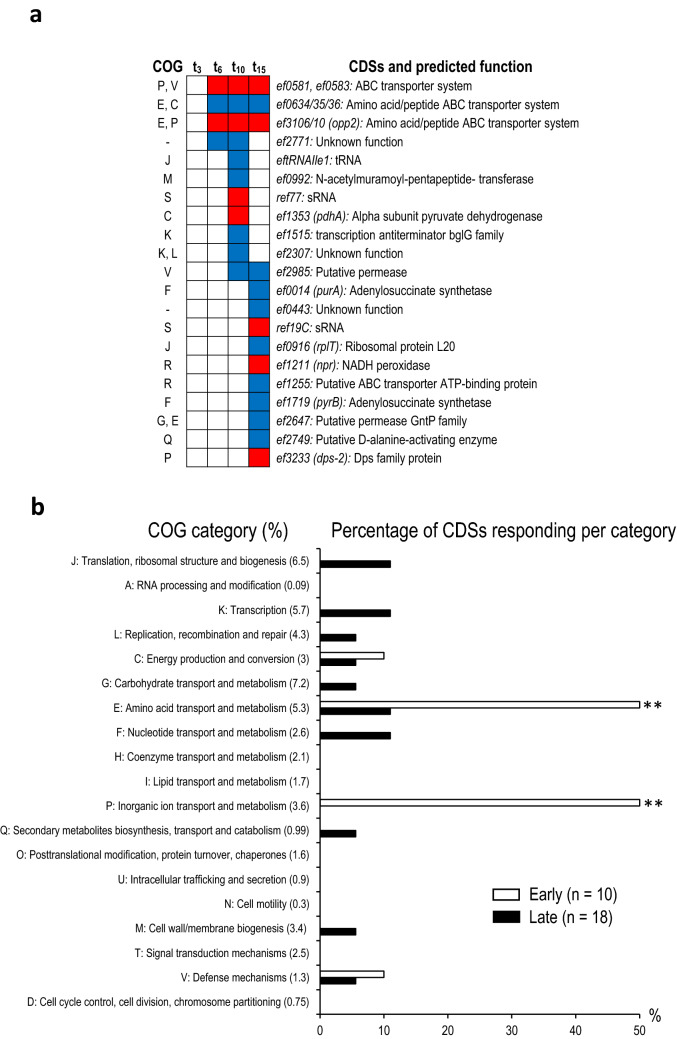
Gene reprogramming during the adaptative response of *E. faecalis* to TCA. (**a**) Early and late responses of CDSs to 0.1% TCA. COG categories are indicated by the letter code of the left of the figure. Significant changes in RNA levels are noted as in Fig. 2a: ‘red’, increased expression; ‘blue’, decreased expression. (**b**) COG functional categories reprogrammed in early and late phase responses. The legend is as in Fig. 2b. Note the coordinated expression of COG categories over time, and the absence of essential or important proteins for growth involved in the response.

In contrast to DCA, *E. faecalis* response to TCA involved significantly fewer genes (Fig. 3a, Tables S4, S5). Overall, the differential gene expression showed a clear timeline revealing a specific response across 28 CDSs. Early changes (t_6_0_) in RNA levels were detected for nine CDSs, including a hypothetical CDS (*ef2771*) of unknown function and the others imbedded in three operonic organizations: *ef0581 and ef0583* (operon *ef0581/84)*, *ef0634 and ef0636* (operon *ef0634/36*), and *ef3107, ef3108, ef3109 and ef3110* (operon *ef3106/10*)*,* predicted to encode transport systems for vitamins, metal ions, amino-acids or peptides^50,51,66,67^. RT-qPCR analyses for those three operons confirmed the RNA-seq conclusions: Compared to t_0_, within the early phase of the adaptation process t_6-0_, RNA levels measured for *ef0581/84* and *ef3106/10* were increased, and decreased for *ef0634/36* (Fig. 3a, Fig. S3 and Table S5). Therefore, the expression of these operons is among the earliest to be modified in response to TCA.

The majority (19) of CDSs, responded during the late phase of the adaptation process (t_15_6_), including *ef0635* (*tyrP*) and *ef3106* that belong to the early responding operons *ef0634/36* and *ef3106/10* (Fig. 3a). Functions assigned to the remaining 17 CDSs suggested that an adjustment of the central metabolism occurred during the late response. This is illustrated by changes in the expression of *pdhA* (*ef1353)* encoding a subunit of the pyruvate dehydrogenase catalysing the formation of acetyl-CoA from pyruvate, and *npr* (*ef1211*), a peroxidase oxidizing the cofactor NADH. Most likely, adjustments in the central metabolism are related to i) variations in the pool management of amino-acids via changes measured during the early response (operons *ef0581/84*, *ef0634/36*, and *ef3106/10*), and the late phase (*ef1255*, encoding a putative tryptophan/tyrosine transporter, and *ef2749,* encoding a putative D-alanine-activating enzyme), and ii) the late changes in the expression of pivotal enzymes involved in the nucleotide metabolism, *purA* (*ef0014*) and *pyrB* (*ef1719*) affecting purine and pyrimidine biosynthesis, respectively, (Table S5)^50,51,66^. This hypothesis is consistent with the sequential expression observed for ‘amino-acid transport/metabolism’ (COG: E) and ‘ion transport metabolism’ (COG: P), which both precede that of ‘nucleotide transport/metabolism’ (COG: F) CDSs, *purA* and *pyrB* (Fig. 3b). This also suggests that genes coordinating the nucleotide pool may be linked to more global regulation modulating amino acid utilization to protein synthesis in response to TCA, as observed by changes in the expression of CDSs involved in the COG category ‘J’ (‘Translation’), *eftRNAIle1* and *rplT (ef0916)*. However, from our data, it is unclear how metabolic pathways involving CDSs of E (amino-acids) and F (nucleotides) COG categories affect the expression of translation related CDSs.

Together, these data reveal that *E. faecalis* is genetically well equipped to deal with TCA, appearing insensitive to this natural compound, possibly due to co-evolution with bile-producing hosts^68^.

### The *ef3106/10* locus encodes an oligopeptide transporter

In less than six minutes, TCA triggered changes in the expression of three predicted transporter systems: *ef0634/36*, *ef0581/84* and *ef3106/10* (Fig. 3a). *ef0634 (tdcA) and ef0635 (tyrP)* are predicted to encode a tyrosine decarboxylase and a permease, respectively, both are involved in tyramine production; *ef0636* (*nhaC-2*) is predicted to encode a Na^+^/H^+^ antiporter of the NhaC family^67,69,70^. *ef0581/84* and *ef3106/10* are both predicted to encode ABC transporter systems, of which the substrate binding protein is predicted to bind peptides, sugars or metal ions^71^. We undertook the functional characterization of the three loci, by constructing in-frame deletion mutants for both individual operons as well as in combination. Compared to the parental strain, with or without 0.1% TCA, no significant growth defects were measured in these mutants; growth similar in rich medium (BHI), under osmotic shock, at low temperatures, at alkali or acidic pHs, or when treated with a series of antibiotics (data not shown). These results indicate that these loci are not essential for growth in rich medium conditions or for response to the stress conditions tested.

Using chemically defined medium (CDM)^72^, in which the concentration of each constituent can be controlled, we demonstrated that the *ef3106/10* operon, named *opp2* (see below), encodes an oligopeptide importer. The parental strain (Wt) and all isogenic deleted mutants grew when tryptophan (W), an essential amino acid for *E. faecalis* (present at 50 µM in CDM), was substituted with W-containing di-, tri- or tetrapeptide (Table 1). Only the ∆*opp2* mutant did not grow when a nonapeptide (Np1) or a decapeptide was provided as a sole W source (Fig. S4). Growth of ∆*opp2* was specifically complemented by the pIL252-*opp2* plasmid carrying the *opp2* locus, but not by the pIL252 vector (Fig. S5). No strain grew when provided with a W-containing 15-mer peptide (Table 1). Our data demonstrate that, in CDM conditions, the *opp2* operon is the unique transporter enabling *E. faecalis* V583 to import peptides larger than 4 and presumably smaller than 15 residues long.

**Table 1:** Import and utilization of W-containing peptides by *E. faecalis* wild type strain and isogenic mutants.

Added to CDM	Wt	∆(*ef0580/4*)	∆(*ef0634/6*)	∆*opp2*	∆*opp* / pIL-opp2	∆*opp* / pIL252
no W	−	−	−	−	−	−
**W** (1)	+	+	+	+	+	+
**VW** (2)	+	+	+	+	*nd*	*nd*
**GW** (2)	+	+	+	+	*nd*	*nd*
**GWG** (3)	+	+	+	+	*nd*	*nd*
**LWMR** (4)	+	+	+	+	*nd*	*nd*
**YPPW** (4)	+	+	+	+	*nd*	*nd*
**NAWVAWDND** (9)	+	+	+	−	+	−
**SFPWMESDVT** (10)	+	+	+	−	+	−
**WWGKKYRASKLGLAR** (15)	−	*nd*	*nd*	−	*nd*	*nd*

The CDM was supplemented with W-containing peptides (first column). The sequence of peptides is provided with the one letter code and length is indicated in brackets. ‘no W’: Absence of W. Strain tested are at the top of each column. Growth was evaluated by measuring the OD_610_ of the culture each 15 min during 20 h (Fig. S3). When growth was observed (‘ + ’), no significant differences between strains were measured. The absence of growth is marked by ‘−’. *nd:* Growth not determined. A representative growth for complementation experiments is provided Fig. S5.

The *opp2* locus compasses the five characteristic CDSs of an ABC transporter system^73^: A substrate-binding protein (Ef3106: Opp2A), two membrane-channel permease proteins (Ef3107/8: Opp2B/2C), and two ATP-binding proteins (Ef3109/10: Opp2F/2D). During the completion of this work, G. Dunny *et col*., reported that two *opp* systems, *opp1* and *opp2* in the *E. faecalis* isolate OG1RF, allow the import of the heptapeptide conjugation pheromone cCF10. These operons correspond to *ef0907/12* and *ef3106/10* in the V583 chromosome^74,75^. Our BLASTp alignments between V583 Opp2 (Opp2A/2B/2C/2D/2F) and OG1RF sequences, showed unique and near identical systems in the two strains (lowest identity for Opp2A: 99%). The high conservation of *opp2* in V583 and OG1RF strains strengthens our conclusion that the *opp2* operon encodes an oligopeptide permease system. Our results also demonstrated that *opp1* and *opp2* have two distinct functions and only *opp2* is able to import nona- and decapeptides and respond specifically to TCA.

### TCA stimulates growth in limiting oligopeptide conditions

The TCA-dependent induction of *opp2* observed in rich medium (BHI) and the essentiality of *opp2* for the peptide intake in CDM (Table 1), prompted us to test whether TCA could modulate peptide utilization. We first established conditions for *opp2*-limited *E. faecalis* growth in CDM by decreasing nonapeptide concentration to maximally couple concentration variations and growth effects. We also used two nonapeptides with different amino acid composition and physicochemical properties, Np1: NAWVAWDND, and Np2: REGHILMWV, to control for any peculiar effect of a nonapeptide on growth (Fig. S6). Results were recorded for nonapeptide concentrations between 1 µM and 5 µM (Fig. S7).

**Figure 4:**
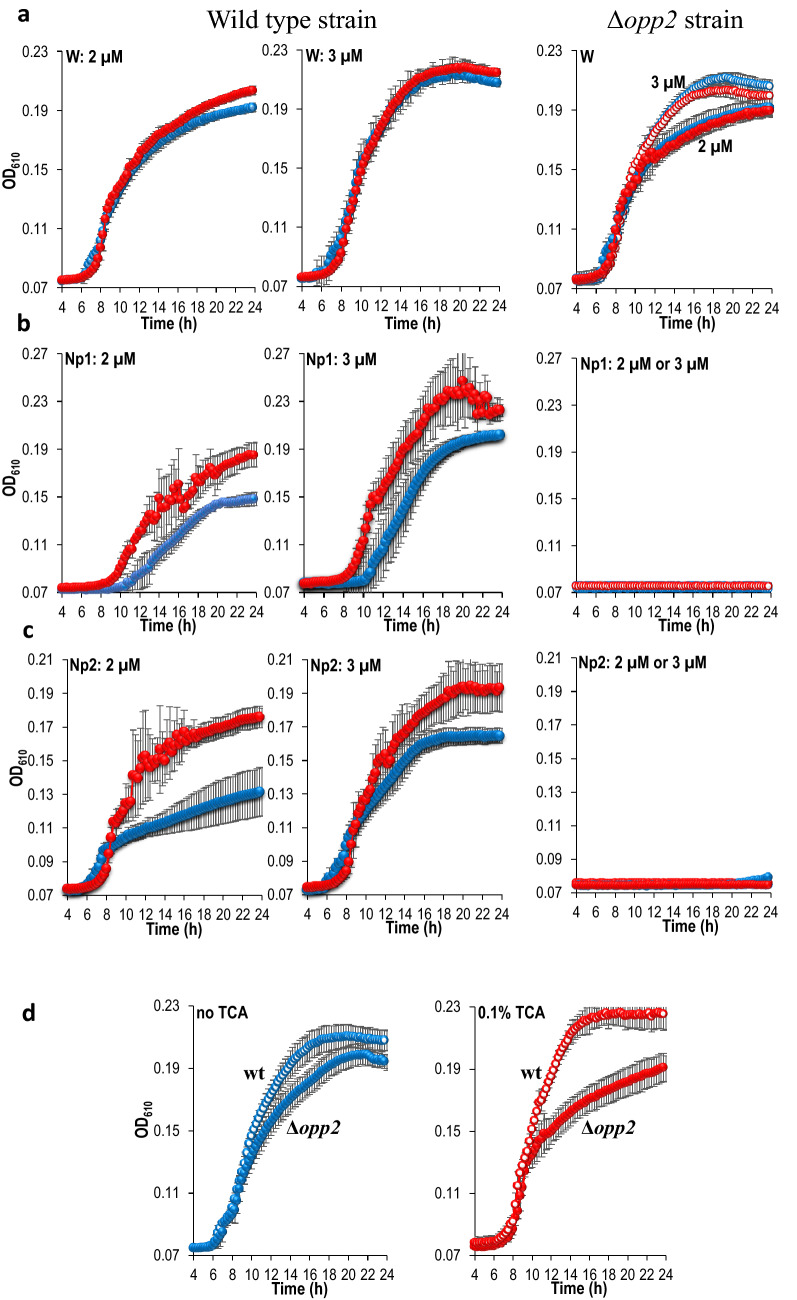
Effect of TCA on *E. faecalis* growth in limiting concentrations of W-containing nonapeptide. (**a,b,c**) From top to bottom, and from left to right, the graphs in the first two columns correspond to the growth of the wild-type strain (wt). The last column shows growth of the Δ*opp2* mutant strain (Δ*opp2*). All growths were performed in CDM medium where W was provided as free amino acid (**a**), or as nonapeptide, NP1: NAWVAWDND (**b**) or Np2: REGHILMWV (**c**). The concentrations are indicated on each graph. For the Δ*opp2* mutant, the same graph shows the growths measured at 2 µM and 3 µM of W-equivalent. **d**: Growth comparison of the wt and Δ*opp2* strains, in CDM medium supplemented with 2 µM W and 3 µM Np2, without TCA (left panel) and with 0.1% TCA (right panel). For all graphs, growth in the absence of TCA is indicated by blue curves; growth in the presence of 0.1% TCA is shown by red curves. Error bars represent the standard deviation of the mean value calculated from at least three independent biological replicates performed in technical duplicates.

Wild type and the ∆*opp2* mutant strains grew similarly in the presence or absence of TCA when W was provided as a free molecule (Fig. 4a), showing that TCA is not involved in the entry of W and has no effect on growth in the absence of the *opp2* locus (Fig. 4a, upper right panel). The growth effect of 0.1% TCA was then tested on the wild-type strain by supplying Np1 and Np2 at 2 µM and 3 µM W-equivalent, in place of free W (Fig. 4b,c). Bacterial growth with Np1 was detected 2 h earlier in presence of TCA, and the entry into stationary phase occurred at higher optical density (OD) when TCA was present (Fig. 4b). 0.3% TCA resulted in slightly more pronounced effects compared with 0.1% TCA, but similar conclusions were reached (Fig. S8). Supplementary Figure S7 shows that 0.1% TCA did not alter the measured ODs and confirms that differences in ODs were due to differences in bacterial densities, not optical artefacts. Using Np2 in identical conditions to Np1, 0.1% TCA enhanced the growth rate and favoured a higher bacterial density in stationary phase (Fig. 4c). The absence of growth with the Δ*opp2* mutant confirmed the strict requirement of the *opp2* locus (Fig. 4b,c, right panels). As anticipated, different growth patterns were observed in the wild type with Np1 and Np2, indicating a peptide sequence-specific effect on *E. faecalis* metabolism, likely the result of nonapeptide compositional and physicochemical differences (Fig. 4b,c and Fig. S6).

To confirm that TCA favoured *E. faecalis* growth in an *opp2*-dependent manner, we compared the growth of the wild type and the ∆*opp2* mutant in CDM supplemented with 2 µM W and 3 µM Np2, in the presence or absence of TCA. In the absence of TCA, both strains grew similarly (Fig. 4d, left panel). In contrast, 0.1% TCA enabled a longer exponential phase and higher stationary phase of the wild type strain compared to the ∆*opp2* mutant (Fig. 4d, right panel).

These results demonstrated that TCA stimulates an ‘overgrowth’ requiring the *opp2*-dependent entry of peptides. The combined effect of *opp2* and TCA may be due to the increased *opp2* expression as observed in the transcriptome analysis, the increased activity of the Opp2 transporter system at the membrane (a target of TCA^76^), and/or the stimulation of a peptide-dependent pathway affecting growth. Therefore, we concluded that TCA favours *E. faecalis* growth by stimulating the uptake or the metabolism of oligopeptides via the *opp2* locus.

Physiological and transcriptomic data indicate that TCA affects the regulation of three transporters, two of which are involved in amino acid metabolism, *ef0634/36 and ef3106/10*. The *ef0634/36* operon encodes enzymes that synthesize tyramine from free tyrosine and expels tyramine^67,69,70^. The TCA-dependent repression of *ef0634/36* may be a way to conserve intracellular tyrosine and leave it available for protein synthesis. In a coordinated manner, the TCA-dependent induction of the *ef3106/10* operon promotes the non-specific entry of amino-acids in peptide form, used in protein synthesis (Table 1, Fig. 4). In doing so, the bacterium directs its entire pool of intracellular amino-acids to protein synthesis. If this hypothesis is correct, the *ef0581/84* operon should also contribute to increased availability of amino-acids.

## Conclusion

**Figure 5:**
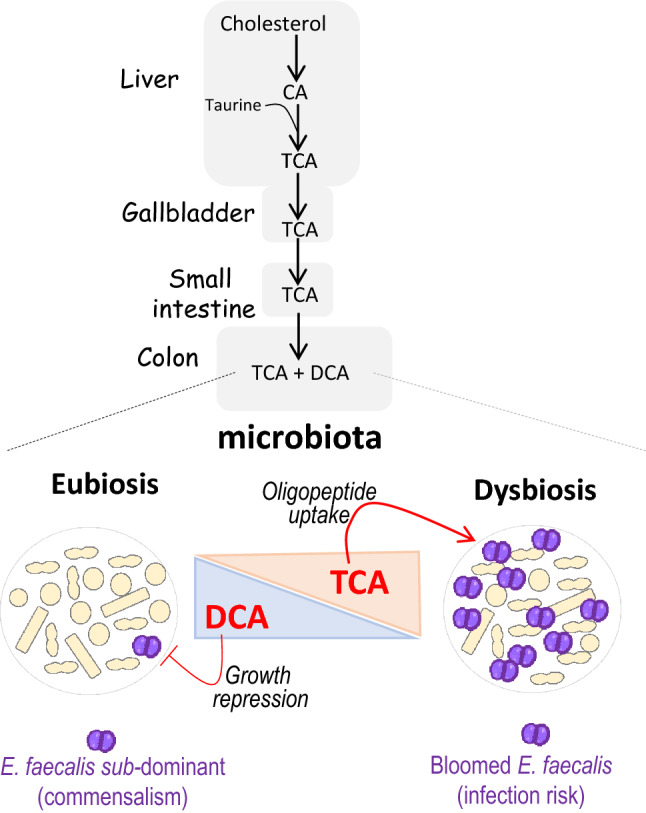
Model of DCA and TCA effects on *E. faecalis* growth in the gut based on in vitro data. DCA and TCA in the gut are BAs synthesized in several steps from hepatic cholesterol. When gut homeostasis is disrupted, the bile composition switches from the predominance of DCA to the predominance of conjugated BAs among which TCA. In vitro*,* DCA hampers *E. faecalis* growth by decreasing the efficacy and the coordination of fundamental processes (translation, transcription, energy production, protein folding and turn-over). This effect could contribute to maintaining the population in a subdominant state during eubiosis. In contrast, TCA enhances oligopeptide intake in limited C- and N-resources, and modulates the amino acid and nucleotide metabolisms, which may favor *E. faecalis* growth during dysbiosis.

We showed here differential sensitivity of *E. faecalis* growth to different BAs. These results should be placed in the context of changes in BA-content accompanying disruption of gut microbiota homeostasis. The sensitivity of *E. faecalis* to BAs in vitro is consistent with in vivo observations. During eubiosis, when primary (CA, CDCA) and secondary (DCA) BAs are prominent, *E. faecalis* growth is severely impeded and the population of the bacterium is sub-dominant. In contrast, during dysbiosis, the prominent conjugated-BA, TCA, is well-tolerated by the bacterium and *E. faecalis* population increases. Our in vitro data indicates that the enteric population of *E. faecalis* tends to reflect the biliary composition of the gut (Fig. 5). At the molecular level, DCA- and TCA-responses exhibit major differences in gene expression reprogramming*.* DCA modified 30 times as many genes than TCA, and substantially slowed growth of *E. faecalis*. This deleterious growth effect can be tightly linked to altered expression of dozens of essential genes. The observation that *E. faecalis* does not appear genetically well-equipped to handle DCA is strengthened by recent studies showing the negative correlation between DCA levels in the gut and the *E. faecalis* population in human and mice^77^. The sub-dominance of *E. faecalis* during eubiosis is likely reinforced by the elimination from the gut of spontaneous BA-resistant mutants^39^. In contrast, TCA had no deleterious effect on *E. faecalis* and triggers a more structured reprogramming, which coordinates amino acid and nucleotide metabolisms, promoting growth in amino-acid limited conditions.

In the gut, dietary proteins provide amino-acids that fuel host- and microbiota anabolism for protein synthesis, and feed carbon, nitrogen and sulfur catabolism for the synthesis of other specific molecules. Proteins undergo hydrolysis in the stomach, generating peptides and oligopeptides, which are further hydrolysed in the small intestine by proteases secreted by enterocytes and representatives of the microbiota. This proteolysis is still active in the large intestine where other microbiota representatives secrete proteases^78^. Free amino-acids in the gut are rare and are primarily absorbed by the host, which may also absorb di-peptides and eventually tri-peptides^79^. Bacteria capable of metabolizing peptides and oligopeptides would doubtlessly evolve to limit competition with the host for amino acid resources. However, the continuous proteolysis creates a bacterial interspecies competition resulting in limiting amounts of oligopeptides; a situation mimicked in vitro in the CDM medium (Fig. 4). Thus, we hypothesize that the induction of *opp2* by TCA helps *E. faecalis* to exploit amino acid resources and favour growth during dysbiosis, when the toxic effect of DCA is absent (Fig. 5).

In summary, we provide here the first snapshot of gene expression reprogramming at sub-generation timeframe of a bacterium sensing and responding to bile acids, and provide clues on changes to bacterial physiology. The gene functions responding to DCA and TCA bring an improved understanding in distinguishing the toxicity of DCA versus the non-toxicity of TCA, suggesting that the bile salt content is one of the players involved in the overgrowth of *E. faecalis* during the disruption of gut homeostasis.

## Methods

### Bacterial strains, gene deletion and complementation

Bacterial strains, plasmids and oligonucleotides are listed in Supplementary Table S6. The *E. faecalis* VE18379 strain, a plasmid free derivative of the clinical strain V583, was used throughout this study^43^. VE18379-derivative strains harbouring in-frame deletions of selected genes were constructed by using the pGhost9 vector as described^80^. VE18379-derivative strains utilized for complementation experiments were constructed by transforming the corresponding parental strain with the empty vector pIL252^81^, or with the derivative plasmid bearing the chromosomal locus including its promoter DNA region, i.e., ~ 300 bp upstream the predicted transcription start site, and ~ 100 bp downstream the predicted transcription terminator^51^ (coordinates provided Table S6). All plasmids constructed were assembled by in vitro isothermal reaction^82^, and *Lactococcus lactis* strain MG1363 (VE14290) was used as recipient. After verification by sequencing, the plasmid constructs were transformed into *E. faecalis* as described^43^. When required, erythromycin was used at a final concentration of 3 µg/mL for *L. lactis* and 100 µg/mL for *E. faecalis*.

### Growth conditions

*E. faecalis* strains were grown in standardized conditions as described^83^. For all experiments, the strain frozen at −80 °C was streak on Brain Heart Infusion (BHI) medium containing 1.5% agar, grown for 20 h at 37 °C and incubated 2 h at room temperature to synchronize the bacteria upon growth resumption^84^. One single colony was inoculated into 5 mL of liquid BHI and grown for 18 h at 37 °C without agitation. The culture was then diluted 1/500-fold in a pre-warmed BHI liquid medium and grown to an optical density at 600 nm (OD_600_) ranging from 0.27 to 0.33 (OD_600_ ~ 0.3), as monitored manually on spectrophotometer at 600 nm weight length (Pharmacia Biotech, Novaspec II). At such a density, the bacterial population ranged from ~ 2 to 4 × 10^8^ cfu/mL, as verified by viable cell counts on BHI plates. This population was then treated specifically according to experimental requirements.

To assay BAs effects in BHI, chemicals were directly added to the culture, and growth was evaluated in a volume of 200µL by measuring OD_610_ on plate reader (TECAN Infinite F2000 PRO). Importantly, to compare the effects of different treatments, each culture used for inoculation was split and each aliquot summitted to the conditions described in the text. BAs were added as an aqueous solution and volumes were adjusted with water, decreasing by 10% the final concentration of media. BAs were used as sodium salts (SIGMA-ALDRICH) at concentrations indicated in the text.

For experiments performed in chemically defined medium (CDM), growth was performed without nucleotides, with 1% casamino-acids, and 0.5% glucose^72^. *E. faecalis* does not grow in CDM without supplemented tryptophan (W) as free amino-acid or W-containing oligopeptide (CLiniSciences, Nanterre, France). Sequences of oligopeptides used are listed Table 1. To set cultures, 1 mL of the culture in BHI described above (OD_600_ ~ 0.3) was spun down at 5,000 rpm for 10 min. Cells were washed twice in 1 mL of 0.9% NaCl solution and recovered in the same volume. Then, successive dilutions were carried out in 0.9% NaCl solution to inoculate the 200 µL CDM at ~ 10^4^ cfu/mL final. Growth was monitored by measuring OD_610_ on plate reader.

### RNA extraction

Individual clones of *E. faecalis* were grown to an OD_600_ ~ 0.3 in BHI medium as described above. A first sample corresponding to the start of the time course experiment (t_0_), was collected before 0.01% final DCA (25 µM) or 0.1% final TCA (2 mM) were individually added. For each culture, samples were then collected at 3, 6, 10 and 15 min after the addition of the BA (noted in the text by t_3_, t_6_, t_10_ and t_15_, respectively). The sampling period was deliberately shorter than the doubling time of the bacterial strain (~ 38 min) in order to analyse changes in the RNA population of bacteria that sensed and responded to the BA; a transcriptome coined ‘evolving transcriptome’^53^. Such a transcriptome identifies more than 90% of the genes involved in an adaptation process and provides clues on the timing of gene expression changes^53,85^. Note that due to the growth impact of DCA, we added an extra time point at 25 min (t_25_) to the experimental course (Table S1). Data were computed considering this extra time point, even if the 15 min evolving transcriptome was sufficient to raise conclusions presented. For this reason, data at t_25_ have not been presented in the ‘Results and discussion’ section: RNAs whose level was changed at t_25_ i) already showed a trend toward a differential expression starting at experimental time points (t_6_, t_10_ or t_15_) preceding t_25_ where they reached the significant statistical threshold; ii) encode functions involved mostly in re-adjusting sugar and lipid metabolisms, iii) make part of operons whose other CDSs were detected at previous time points (Table S2).

Three individual clones (biological replicates) were used for DCA, and four for TCA experiments. Culture samples were spun down during 15 s at 13,000 rpm in 2 mL tubes and pellets were used for total RNA extraction as described^83^. RNA quality was evaluated on Bioanalyzer 2100 (Agilent Technologies) at the @BRIDGE platform (INRAE, Jouy en Josas, France). RNA samples were then utilized for RNA sequencing (RNA-Seq) and qRT-PCR experiments.

### RNA-sequencing and data processing

RNA-seq of DCA treated samples was performed by the MetaGenoPolis company (Jouy en Josas, France) on a SOLiD 5500 W technology instrument (Thermo Fisher Scientific). RNA-seq of TCA treated samples was performed by the contractor company MGX (Montpellier, France) on an Illumina platform (Hi-Seq 2500 technology instrument). RNA-Seq reads were aligned against the genome sequence of the *E. faecalis* strain V583 [GenBank:AE016830.1] using Bowtie (version 1.2.2)^86^ for SOLiD reads and BWA-backtrack (version 0.712-r1039)^16^ for Illumina reads. Gene-level read counts were obtained with Htseq-count (version 0.9.1)^87^ based on the gene annotation of AE016830.1 enriched with non-annotated small RNA encoding genes (sRNAs) previously identified^51^. Statistical analysis of differential expression relied on R library “DESeq2”^88^ and the associated “median ratio method” normalization procedure. Count data are provided in Supplementary Tables S1 and S4 for DCA and TCA, respectively. DESeq2 p-values were converted into q-values using R library “fdrtool”^89^. Genes were called differentially expressed (DEGs) when adjusted p-values (q-values) < 0.05. Differential analyses are summarized in Supplementary Tables S2 and S5 for DCA and TCA, respectively.

### qRT-PCR

To confirm changes in RNA levels observed in response to TCA by RNA-seq, RNA samples used for RNA sequencing were also used for qRT-PCR. Each sample was reversed transcribed using random nonamer oligonucleotides (NONAX) and the SuperScript™ IV reverse transcriptase (ssIV) as described^83^. Total bacterial RNA and the random primer were mixed, incubated for 5 min at 65 °C, and cooled on ice during 3 min. The ssIV was added and the annealing NONAX/RNA was allowed by incubating 5 min at room temperature. Reverse transcription was performed by successive steps of 15 min at 42, 50, 55 and 60 °C. The reaction was stopped by a shift at 85 °C during 5 min. RNA was eliminated by treating the reaction with RNase H (New England Biolabs) during 20 min at 37 °C. Programs ‘Primer 3’ and ‘NormFinder’ were used to design primers and identify genes utilized as internal control, respectively^90^. Based on transcriptomic data, *ef0836* and *ef1093 (ebpC)* mRNAs were selected as internal controls as they did not show significant changes along the experimental time course of the TCA response, and had similar expression levels to RNAs probed *ef0581, ef0634* and *ef3110* (Table S5). The normalization was done using the ‘geNorm’ program^91^. Primer sequences are reported Table S6.

### Cluster of Orthologous Groups and assignment categories based on expression

To analyze and compare functions involved in adaptative responses to DCA and TCA, we utilized the Clusters of Orthologous Groups (COGs) classification of genes^92–94^. This classification does not include small non-protein coding RNAs (tRNAs and sRNAs) and predicted protein-encoding sequences without identified orthology relationships, we incremented the available COG classification for *E. faecalis* (https://www.ncbi.nlm.nih.gov/COG/) by assigning: tRNAs to the ‘J’ category (‘Translation, ribosomal structure or biogenesis’); predicted protein-encoding sequences^50^ and identified sRNAs (*ref* genes described^51^) to the ‘S’ category (‘function unknown’). Based on the known functions of the four ubiquitous sRNAs^95–98^, the following assignments were made: SsrA to the ‘J’ category (‘Translation, ribosomal structure or biogenesis’); SsrS to the ‘K’ category (‘transcription’); RnpB to the ‘A’ category (‘RNA processing and modification’), and Ffs to the ‘U’ category (‘Intracellular trafficking and secretion’). This revisited functional classification is reported Table S3. Enrichment of functional categories was analyzed by computing the percentage of CDSs differentially expressed in a given category among the total differentially expressed during the same time lapse, i.e., early (t_6_0_), and late (t_15_6_). These percentages were then compared to the percentage of CDSs of the corresponding category in the chromosome using Fisher exacts tests on 2 × 2 matrices and adjusted via the Bonferroni correction to account for multiple testing. Statistical significance was considered for an adjusted p-value ≤ 0.05 (Table S3).

## Supplementary Information

Supplementary Information 1.

Supplementary Information 2.

Supplementary Information 3.

Supplementary Information 4.

Supplementary Information 5.

Supplementary Information 6.

Supplementary Information 7.

## Data Availability

The data discussed in this publication have been deposited in NCBI's Gene Expression Omnibus^99^. Transcriptomic raw data (‘.bam’, ‘.bai’ and ‘fastq’ files) are accessible through GEO Series accession number GSE194011 (https://www.ncbi.nlm.nih.gov/geo/query/acc.cgi?acc = GSE194011).
