# Supplementary material for: Adaptation of the gut pathobiont Enterococcus faecalis to deoxycholate and taurocholate bile acids
Source: Sci Rep. 2022 May 19;12:8485. doi: 10.1038/s41598-022-12552-3 (PMC9120511; doi:10.1038/s41598-022-12552-3)
Supplement: Supplementary file 1 — Supplementary Information 1. [file 41598_2022_12552_MOESM1_ESM.docx]

***SUPPLEMENTARY INFORMATION***

**Adaptation of the gut pathobiont *Enterococcus faecalis* to**

**deoxycholate and taurocholate bile acids**

F. Repoila^1,^*, F. Le Bohec^1,#^, C. Guérin^2^, C. Lacoux^1,#^, S. Tiwari^3^, A.K. Jaiswal^3^, M. Passos Santana^1,3^, S.P. Kennedy^4^, B. Quinquis^5^, D. Rainteau^6^, V. Juillard^1^, S. Furlan^1^, P. Bouloc^7^, P. Nicolas^2^, A. Miyoshi^3,^, V. Azevedo^3^, P. Serror^1,^*

**Affiliations:**

^1^Université Paris-Saclay, INRAE, AgroParisTech, Micalis Institute, 78350, Jouy-en-Josas, France.

^2^Université Paris-Saclay, INRAE, MaIAGE, 78350, Jouy-en-Josas, France.

^3^Universidade Federal de Minas Gerais, ICB/UFMG, Minas Gerais, 31270-901, Belo Horizonte, Brazil.

^4^Department of Computational Biology, USR3756 CNRS, Pasteur Institute, 75015 Paris, France.

^5^Université Paris-Saclay, INRAE, MGP, 78350, Jouy-en-Josas, France.

^6^Sorbonne Université, Inserm, Centre de Recherche Saint-Antoine, CRSA, AP-HP, Hôpital Saint Antoine, Biochemistry Department 75012 Paris, France.

^7^Université Paris-Saclay, CEA, CNRS, Institute for Integrative Biology of the Cell (I2BC), 91198 Gif-sur-Yvette, France.

**^#^Present address:**

FLB: Université de Strasbourg, INRAE, SVQV UMR-A 1131, F-68000 Colmar, France; CL: ImmunoSearch, Les Cyclades, Chemin de Camperousse, Grasse, France.

***: For correspondence:** francis.repoila@inrae.fr or pascale.serror@inrae.fr

**Key words:** Bile acid, Gene response, Bacterial adaptation, Oligopeptide import, *Enterococcus* *faecalis*, Intestinal dysbiosis

**Supplementary information contained in this file:**

**Supplementary Tables** (in separated ‘xlsx’ files)

**Table S1.** RNA levels (raw data) measured at each experimental time point during the *E.* *faecalis* response to DCA. The legend is provided in a dedicated sheet in the corresponding ‘.xlsx’ file.

**Table S2.** Differential expression analysis of RNA levels measured during the *E. faecalis* response to DCA. The legend is provided in a dedicated sheet in the corresponding ‘.xlsx’ file.

**Table S3.** Adjusted COG assignments of CDSs in the *E. faecalis* V583 chromosome. The legend is provided in a dedicated sheet in the corresponding ‘.xlsx’ file.

**Table S4.** RNA levels (raw data) measured at each experimental time point during the *E.* *faecalis* response to TCA. The legend is provided in a dedicated sheet in the corresponding ‘.xlsx’ file.

**Table S5.** Differential expression analysis of RNA levels measured during the *E. faecalis* response to TCA. The legend is provided in a dedicated sheet in the corresponding ‘.xlsx’ file.

**Table S6:** Bacterial strains, plasmids and oligonucleotides (‘.xlsx’ file).

**Supplementary Figures** (in this ‘docx’ file)

**Fig. S1.** BA effects on *E. faecalis* growth.

**Fig. S2.** Combined effects of DCA and TCA on *E. faecalis* growth.

**Fig. S3.** Verification by qRT-PCR of changes in RNA levels of early responding operons to TCA.

**Fig. S4.** Growth in CDM supplemented with tryptophan (W) or W-containing nonapeptide (Np1).

**Fig. S5.** Complementation of the *opp2* operon deleted mutant (∆*opp2*).

**Fig. S6.** Major physicochemical differences between nonapeptides Np1 and Np2.

**Fig. S7**. Growth of the *E. faecalis* wild type strain in CDM with different concentrations of nonapeptide (Np1) in presence or absence of TCA.

**Fig. S8.** Effect of 0.1% and 0.3 % TCA on *E. faecalis* growth in limiting concentrations of W-containing nonapeptide (Np1).


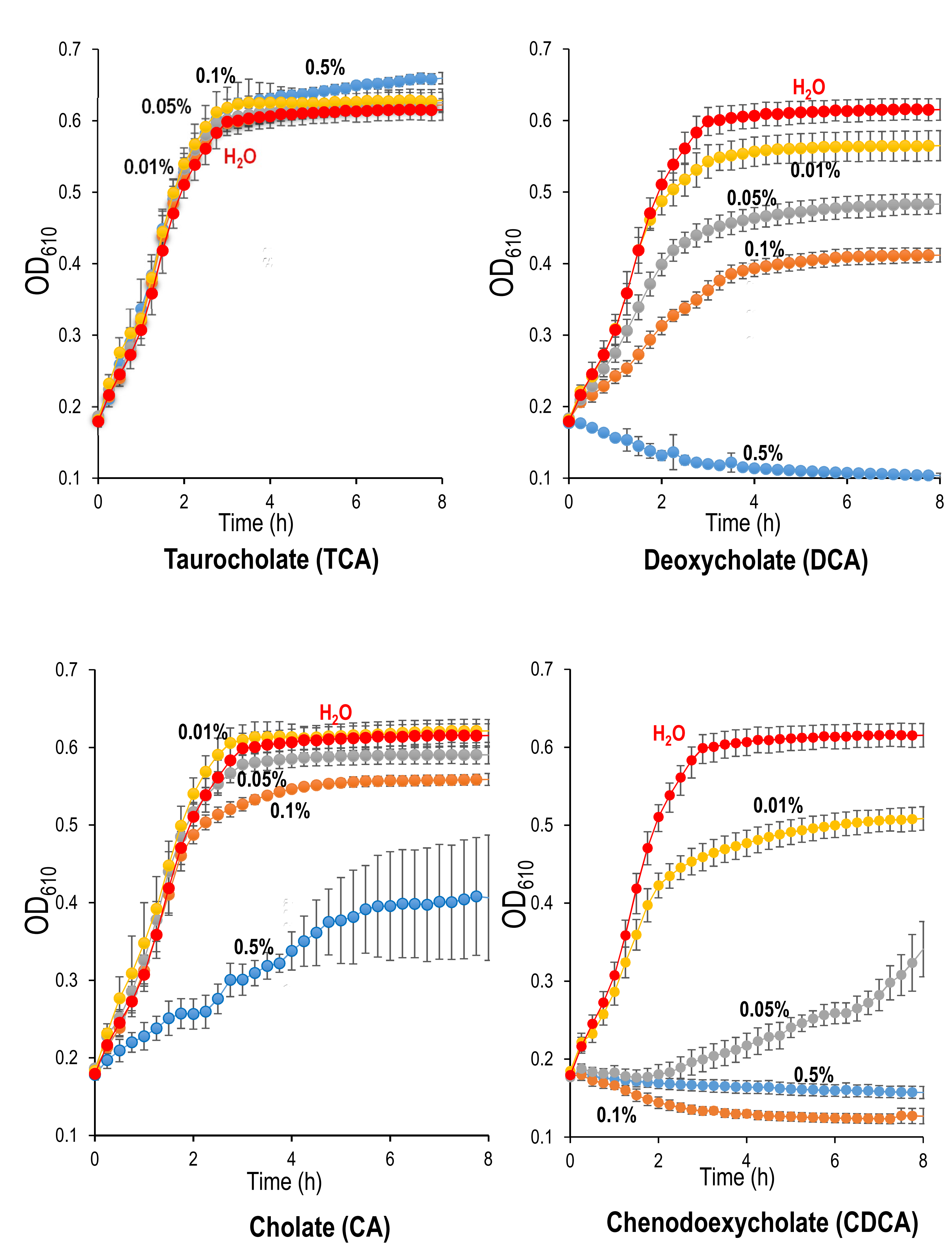


**Fig. S1.** **BA effects on *E. faecalis* growth**. Y-axis: OD_610_ of *E. faecalis* wild type culture in BHI at 37°C (strain VE18379); X-axis: Time in hours (h). Numbers on curves indicate the percentage of BA in the medium. Time 0 corresponds to the addition of the BA to an exponentially growing culture. The control for growth, corresponding to the BA replaced with water, is shown by the red curve (H_2_O). Error bars represent the standard deviation to the mean value calculated from at least three independent biological replicates performed in technical duplicates.

**
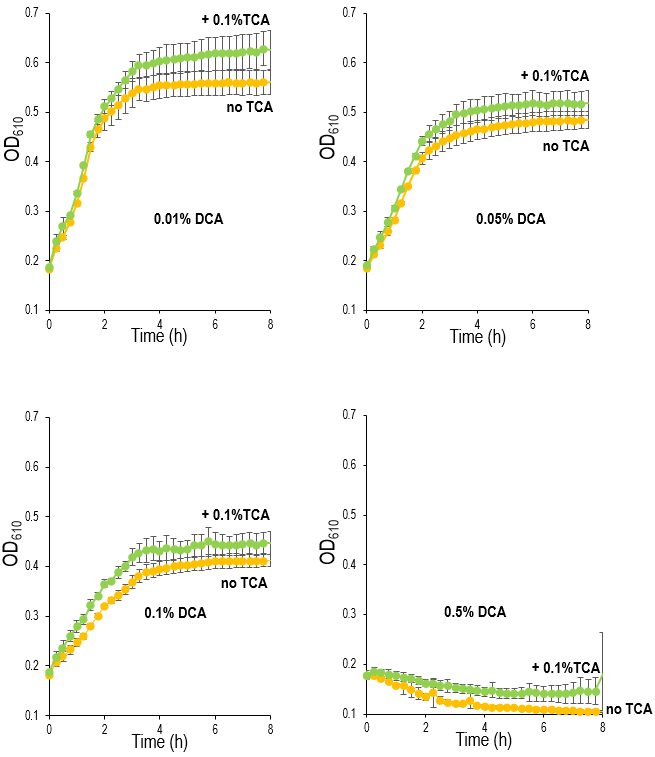
**

**Fig. S2.** **Combined effects of DCA and TCA on *E. faecalis* growth**. Y-axis: OD_610_ of *E. faecalis* wild type culture in BHI at 37°C (strain VE18379); X-axis: Time in hours (h). Time 0 corresponds to the addition of DCA with or without 0.1%TCA, noted by ‘+0.1%TCA’ and ‘no TCA’, respectively. Concentrations of DCA are indicated on each graph. Error bars represent the standard deviation to the mean value calculated from at least three independent biological replicates performed in technical duplicates.


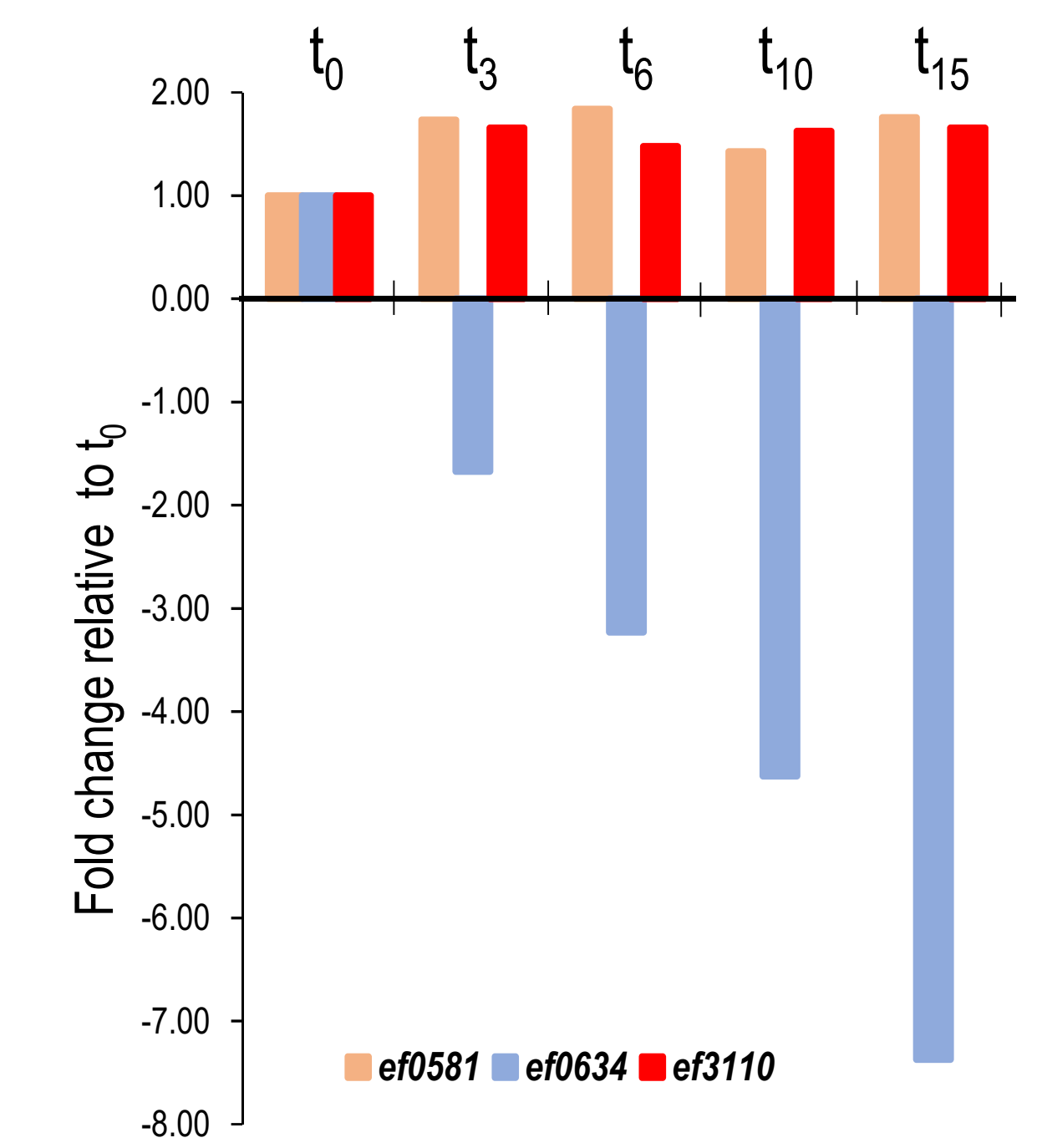


**Fig. S3. Verification by qRT-PCR of changes in RNA levels of early responding operons to TCA.** Changes in RNA levels observed by RNA-seq for operons *ef0580/4*, *ef0634/6* and *opp2*, were verified by RT-qPCR for portions of RNAs corresponding to CDSs *ef0581*, *ef0634* and *ef3110*, respectively. Variations observed parallel those measured by RNA-seq (Fig. 3a, Table S5).


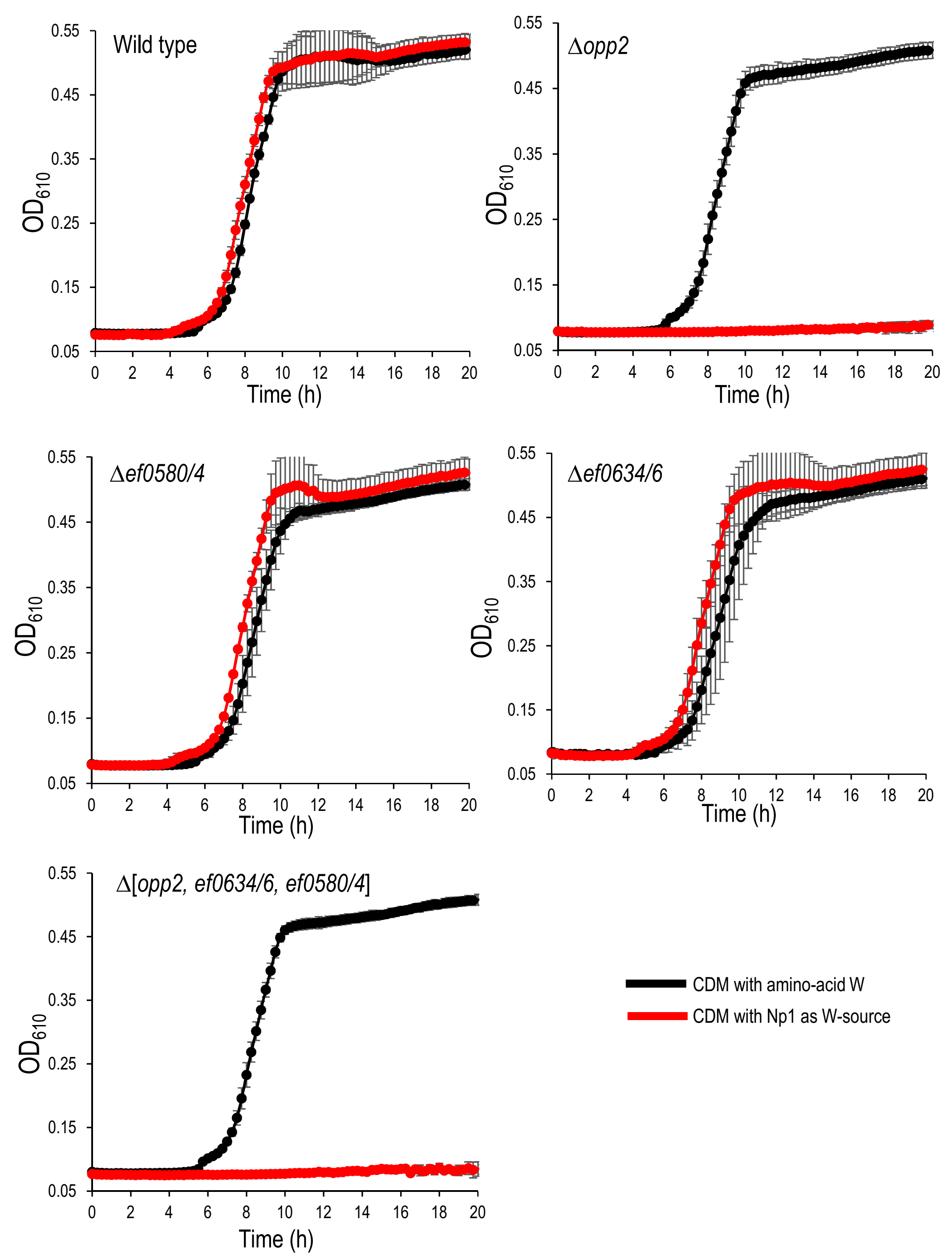


**Fig. S4. Growth in CDM supplemented with tryptophan (W) or W-containing nonapeptide (Np1).** The strain tested is indicated at the top of each panel. Red and black curves show growth in presence of Np1 and W, respectively. Values provided correspond to the average of at least two independent biological replicates performed in technical duplicates. Error bars represent the standard deviation to the mean values.


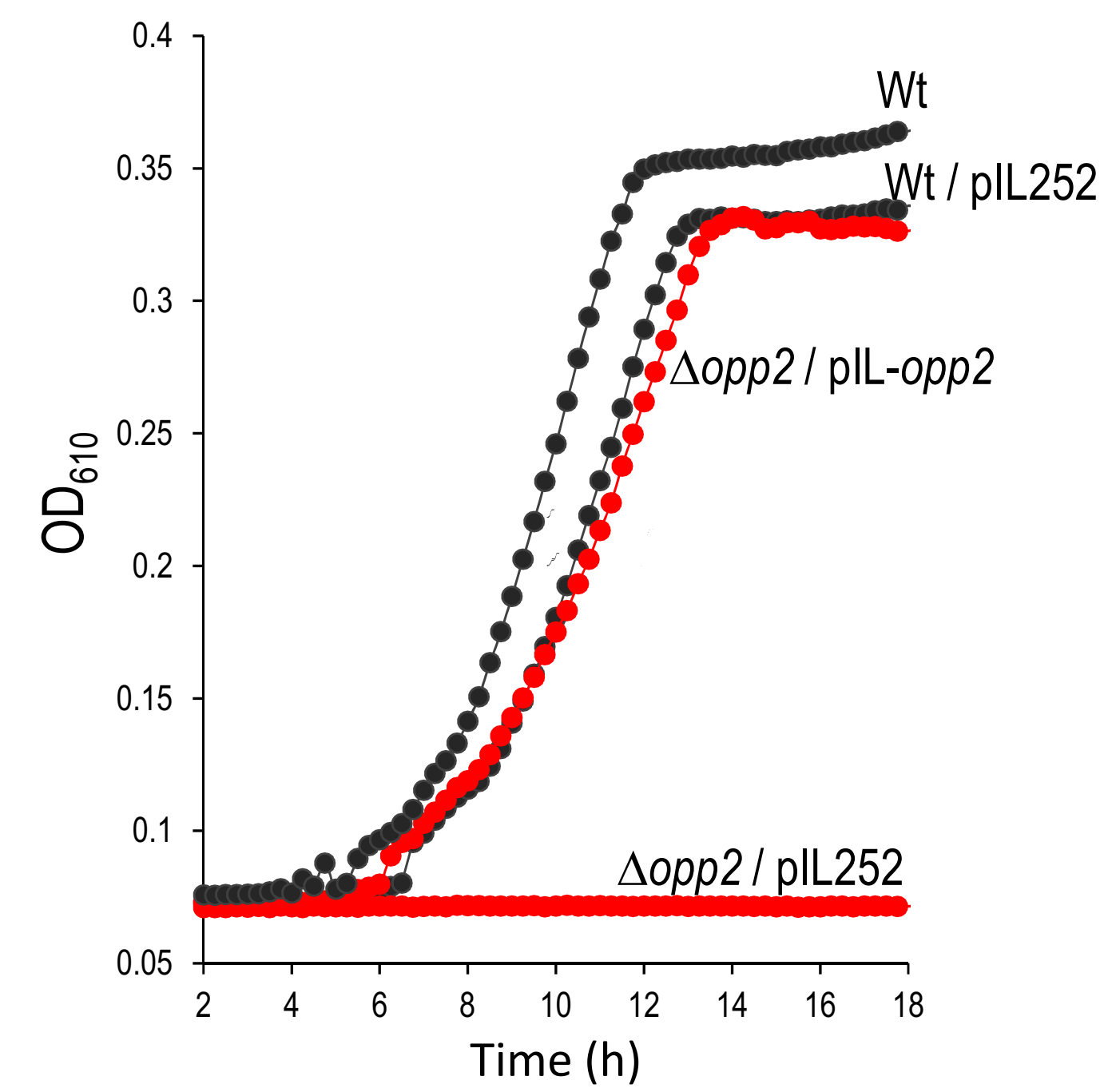


**Fig. S5.** **Complementation of the *opp2* operon deleted mutant (∆*opp2*).** Representative experiment comparing growth of strains Wt, Wt / pIL252, ∆*opp2* / pIL252, and ∆*opp2* / pIL-*opp2* in CDM supplemented with the nonapeptide Np1.


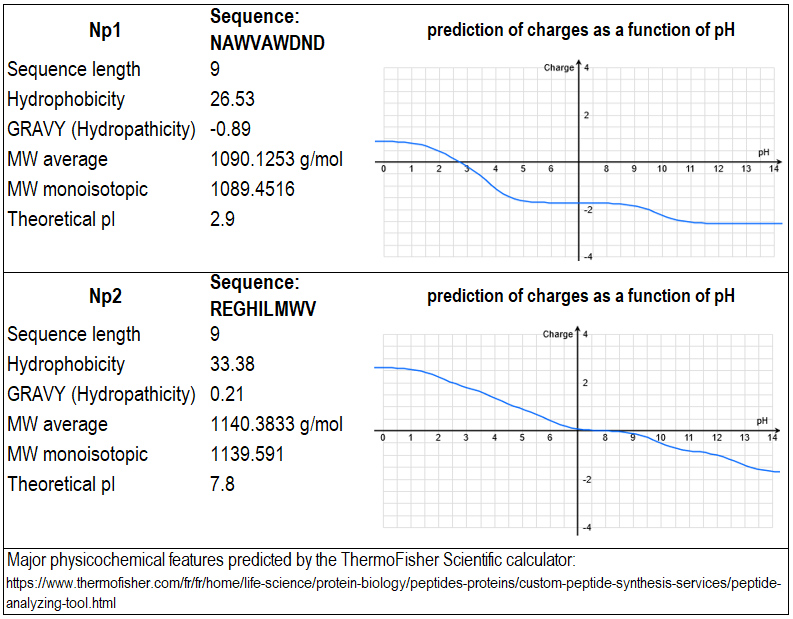


**Fig. S6. Major physicochemical differences between nonapeptides Np1 (NAWVAWDND) and Np2 (REGHILMWV).**


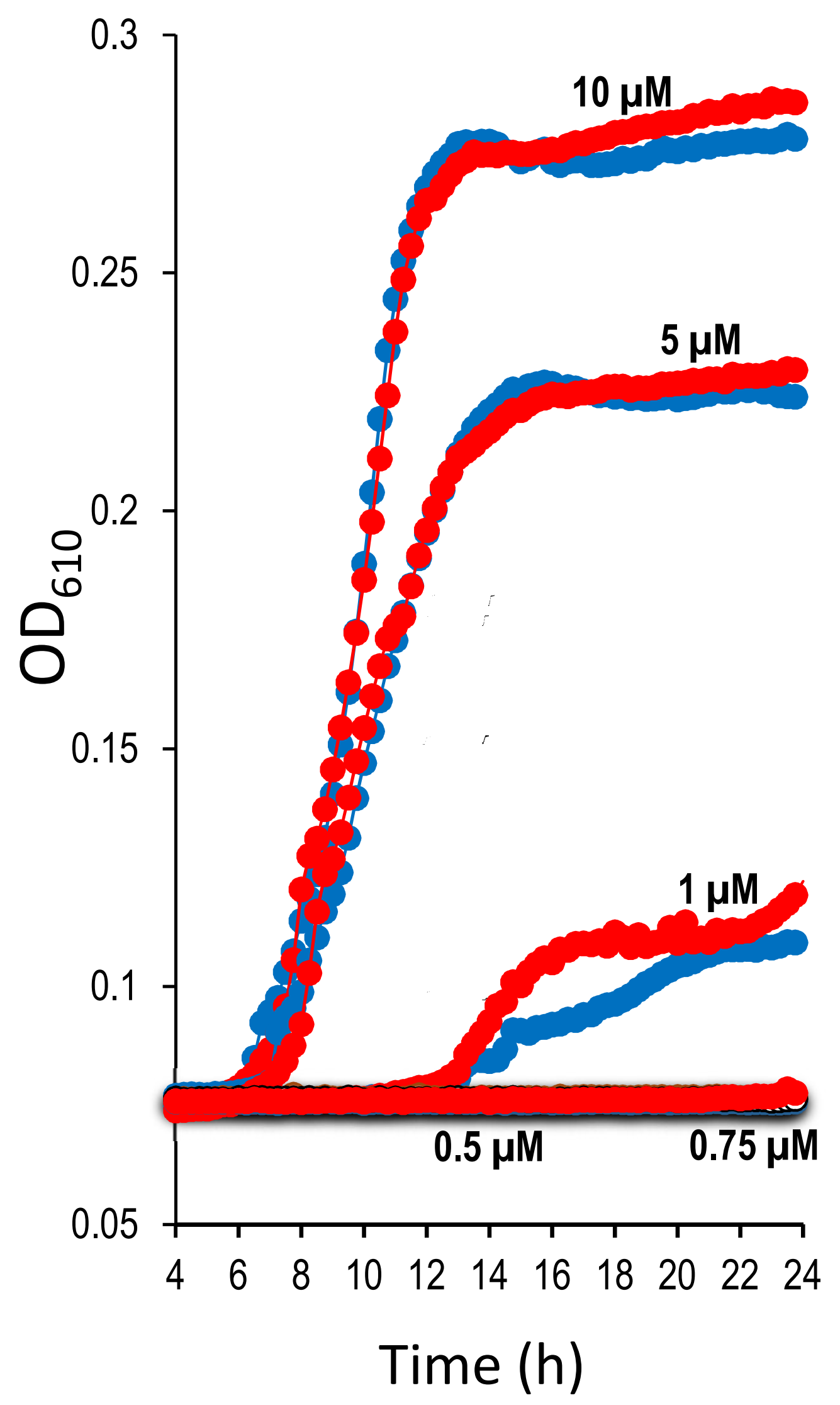


**Fig. S7.** **Representative growth of the *E. faecalis* wild type strain in CDM with different concentrations of nonapeptide (Np1) in presence or absence of TCA.** W-equivalent concentrations provided by Np1 are noted by numbers assigned to curves. Blue curves, growth without TCA; red curves, growth with 0.1% TCA.


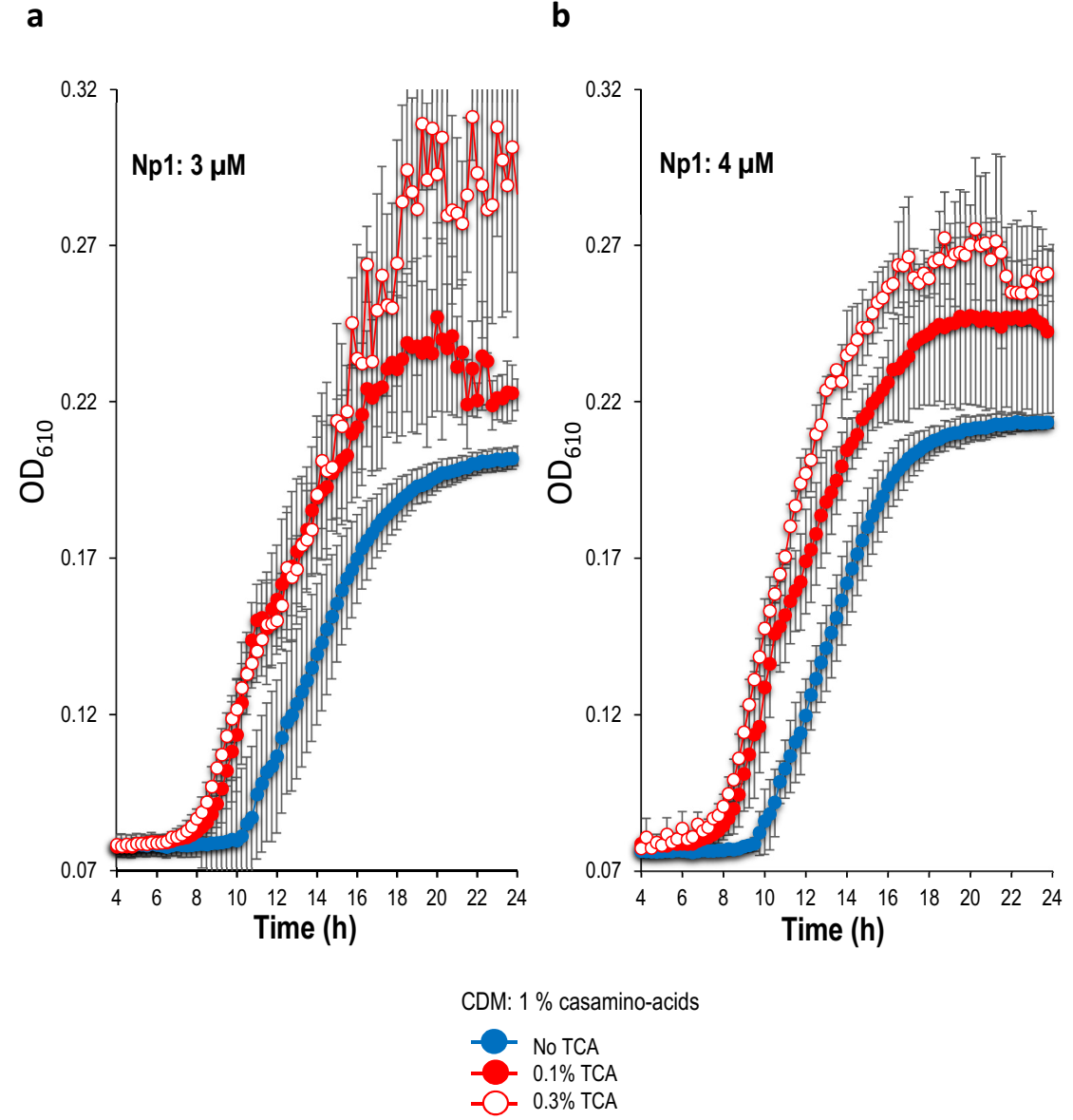


**Fig. S8.** **Effect of 0.1% and 0.3 % TCA on *E. faecalis* growth in limiting concentrations of W-containing nonapeptide (Np1).** W-equivalent concentrations (3 µM and 4µM, panels a and b, respectively) were brought by Np1, in the absence of TCA (blue curves), in the presence of TCA at 0.1% (red curves) or 0.3% (red curves with empty dots). Error bars represent the standard deviation to the mean value calculated from at least three independent biological replicates performed in technical duplicates.
